# On Quickening

**Published:** 1812-07

**Authors:** 


*To the Editors of the Medical and Physical Journal.*

GENTLEMEN,

ALLOW me, through the medium of your useful Journal, to state a few observations in reply to your correspondent Medicus, who has written so learned a dissertation on the vulgar term *Quickening*. After wading through much metaphysical discussion, he comes to this general conclusion, that the medical world, even till now, are ignorant of the cause of this sensation; and, having branded the professors of midwifery with their stumbling over the true cause, finally concludes, that the fœtus has life from its earliest production, and that the sudden motion of the uterus emerging from the pelvis, is the true cause of the term Quickening. Having neither time nor opportunity for entering into discussion on this subject, I wish merely to inform him, that this doctrine has long been the generally-received opinion of of medical men, and that his scholastic dissertation does not contain a single original fact or idea on the subject.

To prove this assertion without occupying many of your valuable pages, I beg to refer him to Dr. Burton's Essays towards a complete System of Midwifery, printed in London more than sixty years ago. If this is not sufficient authority, I shall now refer to a more modern author, viz. Dr. S. H. Jackson's Cautions to Women respecting Pregnancy; second edit. page 53; he says, "its (the uterus) daily increase at length proves the means of releasing it from its confinement, out of which it sometimes will so *suddenly* escape, that the shock it gives will occasion the mother to faint, or grow sick. This sensation she supposes to arise from the act of quickening; and, as this accords tolerably well with the period of the child's real (sensible) motion being sufficiently advanced for her to become sensible of it, the coincidence of the two circumstances occasions her to attribute these symptoms entirely to the entrance of life into the child."

Deeming this sufficient to bear out my assertion, I beg to conclude, by subscribing myself your old correspondent,

CHIRURGICUS.

*Ipswich, June* 3, 1812.

